# Anti-VEGF Treatments and Marketing

**DOI:** 10.22336/rjo.2025.47

**Published:** 2025

**Authors:** Consuela-Mădălina Gheorghe

**Affiliations:** PhD, Philologist, Certified translator

The human body produces a protein called vascular endothelial growth factor, or VEGF. When the body needs new blood vessels, VEGF stimulates their growth and development (Turbert D. Anti-VEGF Treatments. American Academy of Ophthalmology. 26 Jul. 2023. https://www.aao.org/eye-health/drugs/anti-vegf-treatments).

Cells can occasionally create too much VEGF. Thus, the eye may develop aberrant blood vessels as a result. As a result, vision is impaired by these aberrant blood vessels, leading to blindness or low vision (Turbert D. Anti-VEGF Treatments. American Academy of Ophthalmology. 26 Jul. 2023. https://www.aao.org/eye-health/drugs/anti-vegf-treatments).

Anti-VEGF medications decrease the proliferation of blood vessels in the eye by blocking the action of VEGF. This slows down vision loss and prevents or lessens damage from the aberrant blood vessels. It can occasionally even enhance vision (Turbert D. Anti-VEGF Treatments. American Academy of Ophthalmology. 26 Jul. 2023. https://www.aao.org/eye-health/drugs/anti-vegf-treatments).

Ophthalmologists use anti-VEGF medications to treat the following eye conditions: wet age-related macular degeneration (AMD), retinal swelling (macular edema), diabetic retinopathy, and retinal vein occlusion (Turbert D. Anti-VEGF Treatments. American Academy of Ophthalmology. 26 Jul. 2023. https://www.aao.org/eye-health/drugs/anti-vegf-treatments). Among the aforementioned eye conditions, age-related macular degeneration (AMD) is the most prevalent at present (https://www.gminsights.com/industry-analysis/anti-vegf-therapeutics-market).

An aging population and an increase in vision-related conditions like diabetic retinopathy and age-related macular degeneration (AMD) (What are the potential benefits and risks of implementing artificial intelligence in healthcare? - Mains Answer Writing. https://mainsanswerwriting.com/question/97432/) have led to a growing market for anti-VEGF treatment. This market requires a significant investment in real-world data to track brand performance, identify switching patterns, and support commercialization efforts for both new and established drug entrants, including biosimilars and innovative delivery systems.

Through patient access programs in specific markets, marketing efforts emphasize the high costs of these long-term medications, highlight their efficacy, and accommodate patient and doctor preferences regarding dose frequency.

As far as disease prevalence is concerned, the growing incidence of eye disorders associated with high VEGF protein, such as neovascular AMD, diabetic macular edema, and retinal vein occlusion, is driving the market.

One of the main demographic factors driving these treatments is the aging of the world's population.

With new competitors and biosimilars (less expensive versions of existing medications) constantly emerging, the market remains highly competitive.

In 2021, the global anti-VEGF market was estimated to be worth $12.36 billion, and it is expected to continue growing.

The market for anti-VEGF therapies was estimated to be worth USD 13 billion in 2024 and is expected to grow at a compound annual growth rate (CAGR) of 6.1% (Germany: Robert Bosch Venture Capital joins Variantyxs US $26 million Series C funding round. MENA Report. 2021) between 2025 and 2034. Several factors, including increased awareness and a growing demand for treatment, are driving revenue growth in the market. The population's aging and the rise in eye conditions are also factors driving revenue growth (https://www.gminsights.com/industry-analysis/anti-vegf-therapeutics-market).

Regarding the marketing strategies and tools used, ophthalmological organizations use RWD (Real-World Data) to monitor brand usage, patient product switching, and overall market share. RWD is frequently obtained through exclusive collaborations with ophthalmology registries, such as the Romanian Society of Ophthalmology, which collaborates with the American Academy of Ophthalmology to provide Romanian ophthalmologists with access to the comprehensive online educational platform, the ONE Network.

To underscore innovation, marketing places a strong emphasis on advancements in drug delivery methods that reduce the need for injections, such as sustained-release implants and longer-acting injectables. One crucial marketing point is the development of bispecific antibodies (such as faricimab) that target both VEGF and other pathways.

Because these therapies are expensive and require long-term use, marketers may utilize patient access programs to manage expenses and ensure patient access to these therapies.

Thus, focusing on efficacy and safety, marketing highlights how anti-VEGF medications can improve vision in individuals with various illnesses and slow down vision loss.

Some examples of key products include aflibercept (Eylea®) and ranibizumab (Lucentis®). A novel bispecific antibody, called faricimab (VABYSMO®), was developed to extend the interval between injections. Licensed initially for cancer, bevacizumab (Avastin®) is frequently used off-label for eye diseases. As its biosimilar counterparts become available, the pricing dynamic becomes more competitive. Besides the above-mentioned therapeutic solutions, there is also Beovu, which has considerably minimized the number of injections and offers a 12-week dosing interval after the loading phase. Among all these products, it appears that Eylea is the most prominent on the market, with a share of 51.8% in 2024 (https://www.gminsights.com/industry-analysis/anti-vegf-therapeutics-market).

In conclusion, considering the marketing factors in the anti-VEGF market, such as the disease prevalence (age-related macular degeneration, diabetic retinopathy, etc.), competitive landscape, drug innovation (longer-acting injectables and sustained-release implants), real-world data, clinical differentiation (frequency of injections required), cost and value, off-label utilization (that can influence the marketing approaches), and also the key players on the market, hopefully the patients’ health states and quality of life would be improved.

Senior Lecturer Consuela-Mădălina Gheorghe, PhD, Philologist, Certified translator

